# Formation primaquine-5,6-orthoquinone, the putative active and toxic metabolite of primaquine via direct oxidation in human erythrocytes

**DOI:** 10.1186/s12936-019-2658-5

**Published:** 2019-01-30

**Authors:** Pius S. Fasinu, N. P. Dhammika Nanayakkara, Yan-Hong Wang, Narayan D. Chaurasiya, H. M. Bandara Herath, James D. McChesney, Bharathi Avula, Ikhlas Khan, Babu L. Tekwani, Larry A. Walker

**Affiliations:** 1 The National Center for Natural Products Research, The University of Mississippi, University, MS 38677 USA; 2 Department of BioMolecular Sciences, School of Pharmacy, The University of Mississippi, University, MS 38677 USA; 3 Department of Pharmaceutical Sciences, Campbell University, Buies Creek, NC 27501 USA; 4 Ironstone Separations, Inc., Etta, MS 38627 USA; 5 Department of Infectious Diseases, Southern Research Institute, Birmingham, AL USA

## Abstract

**Background:**

The activity and haemolytic toxicity associated with primaquine has been linked to its reactive metabolites. The reactive metabolites are thought to be primarily formed through the action of cytochrome P_450_-mediated pathways. Human erythrocytes generally are not considered a significant contributor to drug biotransformation. As erythrocytes are the target of primaquine toxicity, the ability of erythrocytes to mediate the formation of reactive oxidative primaquine metabolites in the absence of hepatic enzymes, was evaluated.

**Methods:**

Primaquine and its enantiomers were incubated separately with human red blood cells and haemoglobin. Post-incubation analysis was performed with UPLC–MS/MS to identify products of biotransformation.

**Results:**

The major metabolite detected was identified as primaquine-5,6-orthoquinone, reflecting the pathway yielding putative active and haematotoxic metabolites of primaquine, which was formed by oxidative demethylation of 5-hydroxyprimaquine. Incubation of primaquine with haemoglobin in a cell-free system yielded similar results. It appears that the observed biotransformation is due to non-enzymatic processes, perhaps due to reactive oxygen species (ROS) present in erythrocytes or in the haemoglobin incubates.

**Conclusion:**

This study presents new evidence that primaquine-5,6-orthoquinone, the metabolite of primaquine reflecting the oxidative biotransformation pathway, is generated in erythrocytes, probably by non-enzymatic means, and may not require transport from the liver or other tissues.

## Background

The association of primaquine (PQ) with haemolytic toxicity in certain individuals has been recognized since the introduction of the anti-malarial drug over six decades [1]. This toxicity was subsequently determined to be associated with a genetic deficiency in glucose-6-phosphate dehydrogenase (G6PD) [2]. Despite this limitation, PQ has remained in clinical use because of its unique therapeutic indications [3–5]. PQ is used for the radical cure of the persistent liver stages of *Plasmodium vivax* and *Plasmodium ovale* responsible for relapsing malaria [3–5], as well as a prophylactic for all forms of human malaria and as a gametocytocide for transmission interruption in falciparum malaria [6–8]. Despite this long history of clinical use, the mechanisms of biotransformation, efficacy and haemotoxicity are still not fully understood [3–5].

Speculation that a metabolite rather than the parent compound was responsible for haemotoxicity of the members of this class of compounds (8-aminoquinolines) predates the introduction of PQ in 1950s. In 1942, Schönhöfer postulated the possibility of oxidative biodegradation of 8-aminoquinolines such as pamaquine, PQ’s closely related predecessor, to a biologically active quinone [9]. Subsequently, Josephson and co-workers detected a metabolite in droppings of chickens treated with pamaquine which had similar UV absorption spectra, methaemoglobin activity (in chicken erythrocytes) and in vitro anti-malarial activity against *Plasmodium gallinaceum* with that of synthetic pamaquine-orthoquinone, [8-(4-diethylamino-1-methylbutylamino)-5,6-quinolinequinone] [10, 11].

**Fig. 1:**
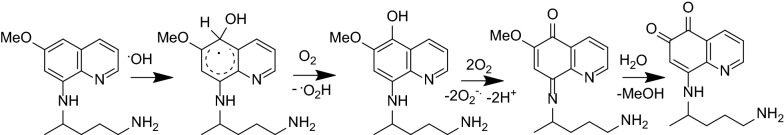
Putative pathway for primaquine metabolism to the PQ-5,6-orthoquinone

The primaquine-derived orthoquinone, 8-(4-amino-1-methylbutylamino)-5,6-quinolinequinone, derived by oxidation of PQ as shown in Fig. 1, is analogous to the pamaquine metabolite, lacking only the diethyl substituents on the terminal amine. Reactive oxygen species (ROS) generated by redox-cycling of products in the pathway leading to PQ-5,6-orthoquinone, have also been proposed to be responsible for the biological activity of PQ [2]. Early in vitro studies have shown accumulation of hydrogen peroxide in erythrocytes when incubated with PQ and suggested the possible formation of a metabolite which can undergo redox-cycling in erythrocytes [12]. In vitro haemolysis studies with PQ also have provided evidence for possible formation of a more active metabolite in erythrocytes and suggested PQ-orthoquinone as the potential metabolite [13].

Several in vitro studies have shown that 5-hydroxy-PQ, 6-demethyl-5-hydroxy-PQ, and PQ-5,6-orthoquinone could form methaemoglobin in erythrocytes at much higher rates than the parent PQ [14–19], and the generation of ROS in erythrocytes by redox-cycling of PQ-5,6-orthoquinone and the corresponding 6-demethyl-5-hydroxy-PQ—or perhaps, the 5-hroxy-PQ and its corresponding quinone-imeine—were suggested as the possible mechanism of this toxicity.

Malaria parasites are also known to be highly susceptible to ROS [20] and generation of ROS has been proposed as the mechanism of action of this class of drug as well as other anti-malarial oxidant drugs [21]. In normal erythrocytes, oxidative defence (glutathione, SOD, and catalase) can neutralize excess ROS preventing haematotoxicity. However, in G6PD-deficient erythrocytes, accumulation of ROS may occur due to impaired oxidative defences leading to haematotoxicity [22, 23]. Even though the pathway leading to PQ-5,6-orthoquinone had been speculated to yield the active and toxic metabolite for a long time [2], it is only recently that it has been positively identified in a mammalian metabolite [19].

CYP 2D6 metabolism of PQ was shown to be required for its radical curative activity in humans [24–27]. A subsequent animal study lends further support to the need for 2D6 metabolism by showing that PQ has very low causal prophylactic activity against sporozoite-induced *Plasmodium berghei* infection in CYP 2D knock-out (KO) mice when compared to wild type (WT) mice or humanized CYP 2D6 (KO/knock-in (KO/KI)) mice [28]. Building on this, Potter and co-workers carried out PQ metabolism studies using WT, KO and KO/KI mice [19]. They found that PQ-5,6-orthoquinone was the major (though short-lived) liver metabolite in WT mice, and that its concentration was reduced to very low levels in KO mice; furthermore, it was restored to about 25% of the WT concentration in KO/KI mice. The activity observed for the above-cited causal prophylactic study [28], was closely correlated with the concentration of the PQ-5,6-orthoquinone generated in the livers of the respective animal groups in the PQ pharmacokinetic (PK) study [19]. This is consistent with the observation that CYP 2D6 metabolism of PQ was required for its radical curative activity [24–26] and probably also for causal prophylactic activity in humans and confirmed PQ-5,6-orthoquinone as reflective of the putative active metabolite. In vitro metabolic studies of PQ with CYP 2D6 further confirmed the formation of PQ-5,6-orthoquinone as a metabolite [29].

However, in a subsequent treatment study of erythrocytic stages using a single dose of PQ, parasitaemia suppression in WT mice was not statistically different from that in CYP 2D KO mice, showing that the genetic deletion of the CYP 2D cluster had no effect on the ability of PQ to eradicate blood stage asexual and sexual parasites [30]. The PQ-5,6-orthoquinone or other PQ-hydroxylated metabolites were not detectable in the plasma of KO mice after oral administration of PQ [19]. This suggests either two different mechanisms for PQ liver- and blood-stage activities, or that the putative active/haemotoxic metabolites related to PQ-5,6-orthoquinone, were formed in the erythrocytes independently of CYP2D6. Previous in vitro studies have suggested the possibility of formation of PQ haemotoxic metabolites in erythrocytes [12, 13].

If active/haemotoxic metabolites of PQ are generated in the red blood cells (RBCs), it may lead to a better understanding of the mechanism of activity and toxicity of PQ, and may enable the design of new 8-aminoquinolines with safer clinical utility. Therefore, the aim of the current study was to evaluate PQ metabolism in human RBCs in vitro, using a stable isotope labelling/tracking method for identification of metabolites, as described previously in detail [31] and to determine kinetics of formation of PQ-5,6-orthoquinone.

## Methods

### Materials

^13^C-labelled PQ (^13^C-PQ, with 6 isotopic carbons in the quinoline nucleus), and PQ-5,6-orthoquinone were synthesized as previously reported [23, 32]. HPLC-grade acetonitrile and methanol were purchased from Fisher Scientific (Fair Lawn, NJ, USA). Water for the HPLC mobile phase was purified in a Milli-Q system (Millipore, Bedford, MA, USA). Human haemoglobin (lyophilized powder), formic acid and glucose were purchased from Sigma-Aldrich (St Louis, MO, USA).

### Preparation of RBC for incubation

Fresh human blood from one single normal (non-G6PD deficient) adult subject was collected into a BD Vacutainer^®^ tube pretreated with EDTA (K2) and refrigerated (4 °C) until ready to use, but not longer than 72 h. Plasma and leukocyte coat were separated and removed after the sample was centrifuged (3000 rpm, 10 min). The erythrocytes were washed in three cycles with NaCl (0.9%). Each cycle of washing involved the vortexing with 2 parts saline, centrifugation (3000 rpm, 10 min) and the removal of the supernatants.

### Incubation of primaquine in erythrocytes/isolated haemoglobin

Racemic PQ and the individual enantiomers (*S*-(+)- and *R*-(−)-primaquine) were incubated separately. For each enantiomer, a 50:50 mix of ^12^C-PQ and ^13^C-PQ with 6 ^13^C incorporation (5 µM) was incubated in 20% haematocrit (erythrocytes diluted with phosphate-buffered saline containing 0.18 M glucose) in a clear 24-well plate at 37 °C under a humidified atmosphere of 95% air and 5% CO2 in an Eppendorf incubator (Hauppauge, NY, USA) attached to a shaker set at 75 rpm. Duplicate samples were monitored for each and all time points. PQ enantiomers were similarly incubated with isolated haemoglobin. Control incubations were performed in the buffer solution without RBCs or haemoglobin.

### Post-incubation analysis

Two different post-incubation treatments were performed on the incubated RBC samples. For the first method (whole RBC treatment), the incubated mixture was precipitated with ice-cold methanol intended to halt metabolic reactions, lyse the erythrocytes and precipitate the erythrocytic proteins. The resulting samples were analysed using UPLC–MS. The second method involved the centrifugation of the incubation mixture (without adding methanol), separating the samples into pellets and supernatants, which were separately analysed. This allowed for much better recovery of protein-bound analytes. The haemoglobin incubations were precipitated with ice-cold methanol prior to centrifugation and the analysis of the supernatants. In preparing for UPLC–MS analysis, samples were diluted with 80% methanol, centrifuged (10,000*g*, 10 min) and the clear supernatants were collected and dried using a Speedvac^®^. The dried samples were re-suspended in 150 µL methanol and centrifuged; clear supernatants were transferred to UPLC sample vials for analysis.

### UPLC/MS metabolite identification

A liquid chromatography-mass spectrometry (LC-MS) method for simultaneous analysis of PQ and its metabolites was developed and employed in this study. The separation of analytes was achieved within a 25-min run time on a Waters ACQUITY UPLC™ BEH Shield RP18 column (100 mm × 2.1 mm I.D., 1.7 µm) equipped with an LC-18 guard column (Vanguard 2.1 × 5 mm, Waters Corp, Milford, MA, USA) using an ACQUITY UPLC system (Waters Corp, Milford, MA, USA). The UPLC system includes binary solvent manager, sampler manager, column compartment, and photodiode array (PDA) detector. The mobile phase, consisting of water with 0.05% formic acid (A) and acetonitrile with 0.05% formic acid (B), was applied at a flow rate of 0.3 mL/min in the following gradient elution: 0–5 min, 1% B; 5–14 min, 1% B to 13% B; 14–18 min, 13% B to 25% B; 18–22 min, 25% B to 33% B; 22–23 min, 33% B to 43% B; and, 23–25 min, 43% B to 100% B. Each run was followed by a 3-min wash with 100% B and an equilibration period of 3.5 min with 99% A/1% B. Column and sample temperatures were set at 50 and 15 °C, respectively. Injection volume was 10 µL, and the strong needle wash (90/10; acetonitrile/water, v/v) and weak needle wash solution (10/90; acetonitrile/water) were used. Peaks were assigned with respect to the mass spectra and retention time of reference compounds or tentatively identified on the basis of high resolution accurate mass.

Mass spectrometric analyses were performed using electrospray ionization (ESI) in positive mode on a Waters Xevo G2-S QToF mass spectrometer (Waters Corp, Manchester, UK). The MS instrument was operated in the following conditions: mass scan range of 50–1200 Da, capillary of 3.0 kV, cone of 30 V, source temperature of 80 °C, desolvation temperature of 450 °C, desolvation gas flow of 800 L/h, cone gas flow of 50 L/h, and a collision energy of 6 eV. Leucine-enkephalin was used for the lock mass at a concentration of 5 ng/mL and flow rate of 10 µL/min. Ions [M + H]^+^ (m/z 556.2771 Da) and fragment ion (m/z 278.1141 Da) of leucine-enkephalin were applied to ensure mass accuracy during the MS analysis. The lock spray interval was set at 30 s, and the data were averaged over three scans. The mass spectrometer was programmed to step between low (10 V) and elevated (10–45 V) collision energies on the gas cell, using a scan time of 0.5 s per function.

Metabolites in the accurate mass data were found using the MetaboLynx^®^ software. The data were searched using predicted metabolite mass, mass defects, isotope, and fragmentation patterns. Particularly helpful was scanning for the isotope (+6 mu) peaks resultant from the use of a mixture of equal quantities of ^12^C and ^13^C PQ. Each sample was subjected to data acquisition in full scan and data-dependent positive MS/MS, targeted MS/MS (ESI positive ionization mode) and high-resolution MS (HRMS) modes using the Waters ACQUITYTM XEVO QTOF Mass Spectrometer (Waters Corp, Manchester, UK) connected to the UPLC system via an ESI interface. Identification of metabolites was assisted by their HRMS data, which were used to calculate their elemental compositions. The full scan mass data were screened and filtered using Waters MetaboLynx XS software. The qualitative metabolite identification was performed using this software package.

### Quantitative analysis of PQ and PQ-5,6-orthoquinone

Reference standards of PQ and PQ-5,6-orthoquinone were dissolved in water and methanol, respectively. Cell-free media, made of phosphate buffered saline, was spiked with PQ and PQ-5,6-orthoquinone solutions in triplicate to final concentrations between 0.5 and 10 µg/mL (using 6 concentration points). The spiked solutions were treated similarly as the incubation mixtures and were analysed with UPLC/MS. A calibration curve was generated with a linear equation (R^2^ > 0.9), and was used to determine the concentrations of PQ and PQ-5,6-orthoquinone in the incubation mixtures. Qualitative detection of PQ-5,6-orthoquinone in haemoglobin incubations was done by comparison of LC-MS/MS profile with synthetic PQ-5,6-orthoquinone.

## Results

**Fig. 2:**
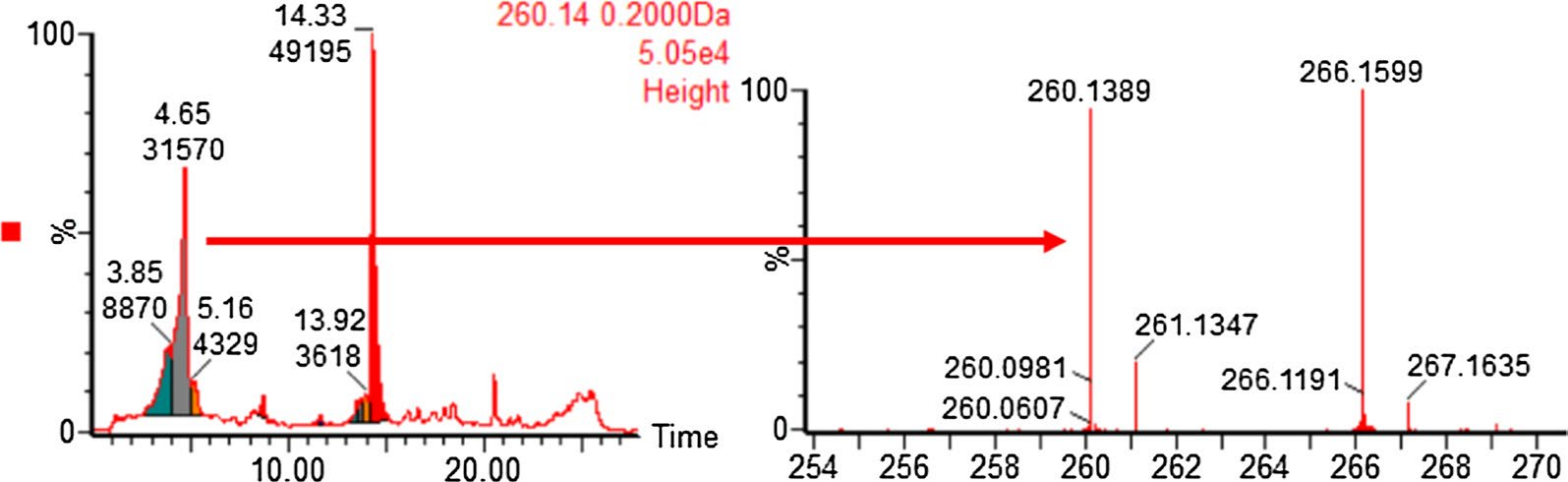
LC/MS chromatogram showing orthoquinone and PQ after incubation of human RBCs with PQ containing a mixture of 12C-PQ and 13C-PQ (13C at 6 quinoline core carbons). The peak at retention time 4.65 min corresponds to the orthoquinone standard, while the peak at 14 min corresponds to PQ. The mass spectrometric fragmentation at right shows the expected +6 mu

A substantial amount of PQ-orthoquinone was detected in the RBC incubates of racemic PQ and the individual enantiomers, with a rapid disappearance of PQ within the first hour. PQ orthoquinone was detected in haemoglobin incubation after 30 min with no further observable time-dependent changes. The metabolites were tracked by following the detection of paired fragments (+6 mu apart). The identity of the PQ-orthoquinone was confirmed on the basis of predicted mass, observed retention time and comparison with synthetic standard (Fig. 2). Both enantiomers of PQ readily gave rise to the PQ-orthoquinone, and no significant difference was observed.

**Fig. 3:**
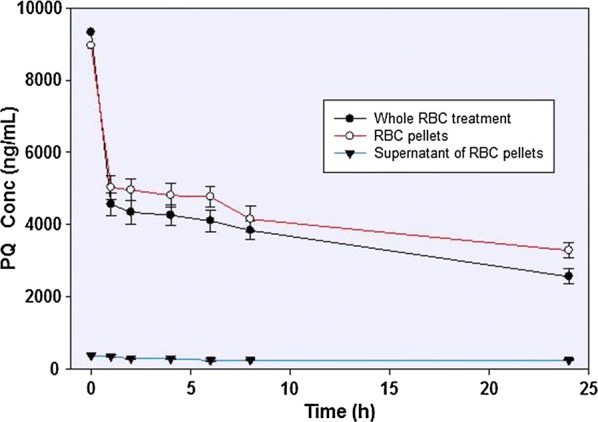
The concentration-time profiles of primaquine incubated with human RBC. Whole RBC treatment involved the precipitation and lysing of the cells followed by recovery of the analytes. Alternatively, the incubation mixture was separated into the cellular pellets and the supernatants prior to analyte recovery and analysis

In the erythrocyte incubations (with PBS, pH 7.4), the majority of the PQ is rapidly partitioned into the red cell fraction, with much reduced concentrations in the medium. The majority of the conversion of PQ to PQ-orthoquinone is observed already at 1 h (with up to 50% substrate depletion) in the red cell fraction, and further incubation for up to 24 h showed only a slow further PQ depletion (Fig. 3).

**Fig. 4:**
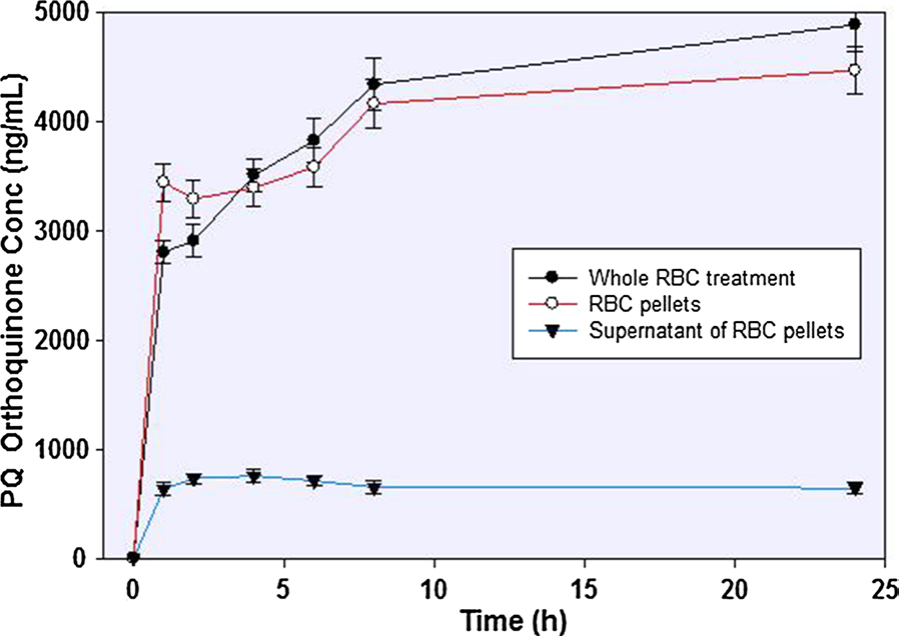
The concentration-time profile of primaquine 5,6-orthoquinone formed from primaquine incubation in human RBC. Whole RBC treatment involved the precipitation and lysing of the cells followed by recovery of the analytes. Alternatively, the incubation mixture was separated into the cellular pellets and the supernatants prior to analyte recovery and analysis

More than 75% of the depleted PQ was converted to PQ-orthoquinone (Fig. 4). The appearance of the PQ-orthoquinone tracks closely the disappearance of the parent drug. Small amounts of other metabolites could be observed, but these could not be unequivocally identified or quantified.

The recovery of the metabolites from the precipitated and lysed RBCs confirms that these reactions are intracellular, although it appears that a fraction of the PQ-orthoquinone does appear in the medium. Control incubations using buffer alone (without adding RBC or haemoglobin) run under similar incubation conditions did not yield any metabolites.

## Discussion

A majority of the primary metabolites generated by PQ in human and animal species are viewed as deriving from three distinct pathways: first, the oxidative deamination of the side chain mediated by the sequential activity of mono-amine oxidase and aldehyde dehydrogenase generates carboxyprimaquine as a principal metabolite [33–36]; second, hydroxylated metabolites formed by ring oxidation, especially through the activity of CYP2D6 [19, 28, 29]; and thirdly, direct conjugation with glucuronide, glucose, carbamate or acetate [37]. Unlike most other cells, mature erythrocytes are anucleated, lacking ribosomes and mitochondria, and are thus incapable of protein synthesis. RBCs are equipped with robust metabolic systems to maintain cellular energy supply, and enzymes for the protection of its vital components from harmful oxidative reactions, but are not known to contain substantial activities analogous to the drug metabolizing CYPs [22, 38].

However, several studies have shown that RBCs may contribute to the metabolism of endogenous and exogenous substances including drugs [38, 39]. While RBCs lack the capacity for conjugation reactions like glucuronidation and sulfation, RBCs’ capability for other conjugation reactions, including acetylation, methylation and glutathione addition, has been reported [39, 40]. Enzymatic oxidation of drugs has not been reported in erythrocytes. However, bioreduction of some compounds has been reported [41, 42].

This study unequivocally identifies, for the first time, the capacity of erythrocytes to transform PQ to PQ-5,6-orthoquinone and it accounted for more than 75% of depleted PQ. It appears that the initial hydroxylation of the quinoline ring of PQ in RBCs may well be caused by non-enzymatic radical reactions. Apparent continuation of biotransformation even after addition of methanol into incubation mixtures, and the observed equal rates and amounts of PQ-orthoquinone formation for both PQ (*S*)- and (*R*)-enantiomers (indicating non-stereospecific nature of the biotransformation), support the argument that the initial hydroxylation was not enzymatic but radical (Fig. 1). In previous studies with CYP 2D6 enzyme, different ring-hydroxylation profiles for PQ enantiomers was reported, and additionally, the rate of formation of PQ-orthoquinone for (*S*)-PQ was roughly twice that for (*R*)-PQ [29]. This enzyme-dependent and preferential PQ oxidation of the enantiomers was not observed in the current study.

Oxyhaemoglobin undergoes spontaneous autoxidation yielding superoxide radicals and methaemoglobin [22, 38]. Superoxide dismutase in RBCs converts superoxide radicals to hydrogen peroxide, which reacts with ferrous ions to form highly reactive hydroxyl radicals. Even though a bulk of these ROS is neutralized by oxidative defence mechanisms, they tend to oxidize compounds such as unsaturated fatty acids in erythrocytes [43].

Hydroxyl radicals are also known to hydroxylate quinolines [44, 45] and other aromatic compounds [46–48] even under physiological conditions [47, 49–51]. PQ, an 8-aminoquinoline with several structural points susceptible to oxidation, especially at the 5-position [52], may undergo hydroxylation in the intra-erythrocytic environment. The rate of hydroxylation of the quinoline ring would depend on the ROS concentration in RBCs. Even though ROS concentration in normal RBCs is low, parasitized [20, 53] and G6PD-deficient [21] RBCs are known to contain elevated levels of ROS. Due to the lack of other degradative enzymes, such as MAO in RBCs, accumulated PQ may persist and be susceptible to undergo slow quinoline ring hydroxylation the 5-position [52]. It has been shown that 5-hydroxyPQ is highly unstable under neutral conditions and spontaneously undergoes oxidation to form the quinone-imine, with further breakdown to 6-demethyl-5-hydroxy-PQ and PQ-orthoquinone [19]. These latter, and perhaps also quinone-imine/5-hydroxy-PQ, can undergo redox-cycling, generating ROS. This process will accelerate the accumulation of ROS especially in parasitized and G6PD-deficient RBCs [20, 22, 53]. ROS will kill plasmodial parasites which are known to be highly sensitive to externally generated ROS [20, 21, 53]. Even though the elevated level of drug-induced ROS can be neutralized by the oxidative defence mechanism in normal RBCs, the weakened oxidative defence mechanism of G6PD-deficient erythrocytes may be overwhelmed, triggering methaemoglobinaemia, erythrocyte damage and subsequent spleenic removal resulting in clinical anaemia [22].

It has been suggested that the reactive metabolites of PQ are generated in the liver and transported to the RBCs prior to clinical manifestation of haemolytic toxicity. PQ-induced methaemoglobinaemia and haemolysis occur at least 24 h after the drug administration [2]. PQ PK studies in mice [19], have suggested that PQ-5,6-orthoquinone is formed extremely rapidly in the liver, and disappeared from the circulation very rapidly. In a single-dose PQ PK studies in humans, PQ-5,6-orthoquinone was not detectable in plasma [36]. The results of this study support an alternative explanation that formation of the redox active PQ metabolites in RBCs followed by progressive oxidative stress may be responsible for PQ-induced haemolysis.

The formation of the PQ-5,6-orthoquinone as a major metabolite in RBCs is particularly revealing because of its strongly suspected link to the haemolytic response as well as blood-stage antiparasitic activity. This may also explain the observation that the CYP2D cluster was not required for PQ to eradicate the blood-stage asexual and sexual (*P. berghei)* parasites in mice [30]. These results will enhance an understanding of PQ metabolism and the contribution of RBC in this regard. It will also improve appreciation of how PQ can cause haemolysis without the contribution of liver metabolism. This understanding may enable the design of drug candidates with better therapeutic index.

## Conclusion

This study presents new evidence to show that oxidative metabolites of PQ can be formed in erythrocytes. The major erythrocytic metabolite identified in this study, PQ-5,6-orthoquinone, has been suggested to reflect the oxidative pathway responsible for blood-stage antiparasitic activity and haematotoxicity of primaquine. Further studies involving human subjects should include analysis of red blood cells, in addition to plasma and urine, for relevant metabolites.

## Abbreviations

CYP: cytochrome P450
G6PD: glucose-6-phosphate dehydrogenase
LC–MS: liquid chromatography tandem–mass spectrometry
PQ: primaquine
RBC: red blood cells
UPLC–MS: ultra-performance liquid chromatography tandem–mass spectrometry
